# Herbal medicine use in the districts of Nakapiripirit, Pallisa, Kanungu, and Mukono in Uganda

**DOI:** 10.1186/1746-4269-8-35

**Published:** 2012-09-03

**Authors:** John RS Tabuti, Collins B Kukunda, Daniel Kaweesi, Ossy MJ Kasilo

**Affiliations:** 1 Makerere University, College of Agricultural and Environmental Sciences (MUCAES), P.O. Box 7062, Kampala, Uganda; 2 Department of Botany, Herbarium and Botanic Garden, Makerere University, P. O. Box 7062, Kampala, Uganda; 3 Uganda National Commission for UNESCO, Ministry of Education & Sports, Embassy House, King George VI Way, Kampala, Uganda; 4 World Health Organization, WHO Regional Office for Africa, P.O. Box 6, Brazzaville, Republic of Congo

**Keywords:** Ethnomedicine, Traditional medicine, Health seeking behaviour

## Abstract

**Background:**

Traditional medicine (TM) occupies a special place in the management of diseases in Uganda. Not with standing the many people relying on TM, indigenous knowledge (IK) related to TM is getting steadily eroded. To slow down this loss it is necessary to document and conserve as much of the knowledge as possible. This study was conducted to document the IK relevant to traditional medicine in the districts of Mukono, Nakapiripirit, Kanungu and Pallisa, in Uganda.

**Methods:**

An ethnobotanical survey was conducted between October 2008 and February 2009 using techniques of key informant interviews and household interviews.

**Results:**

The common diseases and conditions in the four districts include malaria, cough, headache, diarrhea, abdominal pain, flu, backache and eye diseases. Respondents stated that when they fall sick they self medicate using plant medicines or consult western-trained medicine practitioners. Self medication using herbal medicines was reported mostly by respondents of Nakapiripirit and Mukono. Respondents have knowledge to treat 78 ailments using herbal medicines. 44 species, mentioned by three or more respondents have been prioritized. The most frequently used part in herbal medicines is the leaf, followed by the stem and root. People sometime use animal parts, soil, salt and water from a grass roof, in traditional medicines. Herbal medicines are stored for short periods of time in bottles. The knowledge to treat ailments is acquired from parents and grandparents. Respondents’ age and tribe appears to have a significant influence on knowledge of herbal medicine, while gender does not.

**Conclusion:**

This survey has indicated that IK associated with TM stills exists and that TM is still important in Uganda because many people use it as a first line of health care when they fall sick. Age and tribe influence the level of IK associated with herbal medicine, but gender does not.

## Introduction

Traditional medicine (TM) has been used by humans for thousands of years. The World Health Organization (2002), defines traditional medicine, in part, as a medicine system that includes medication therapies like herbal medicines as a well as non-medication therapies like acupuncture. The same organization defines herbal medicines to include herbs, herbal materials, herbal preparations and finished herbal products, that contain as active ingredients parts of plants, or other plant materials, or combinations thereof. The World Health Organization (WHO) estimates that 80% of the population living in developing countries uses TM for their primary health care needs [1]. However, this percentage varies from country to country. For instance, 90% of the population in Ethiopia, 70% in Rwanda, and 60% in Uganda and Tanzania use TM for their PHC [2]. TM is widely used in Uganda for the prevention, diagnosis and treatment of social, mental and physical illness [3]. Although a diversity of material – plant, animal and inorganic material – are used in traditional medicines, plants dominate.

In Uganda, households possess indigenous knowledge of traditional cures for non complicated ailments. On the other hand, Traditional medicine practitioners (TMPs) are an invaluable source of specialized knowledge about TM and are very important human resources for the practice and delivery of primary health care services [3]. The WHO recognizes the invaluable role of TM and its practitioners, and it is for this reason that the Alma Ata Declaration of 1978 recommended that TM and its practitioners should be integrated into primary health care programmes [4] as important resources for achieving health for all.

Although a majority of people rely on TM, indigenous knowledge (IK) related to TM is getting steadily eroded[ [5]. It is believed that this is a consequence of people adopting new lifestyles and migrating to urban centers [6]. Other workers have identified lack of confidence among users and practitioners as a cause of loss of knowledge of TM [7,8]. In the African Region, IK is handed down from generation to generation by oral tradition, and sometimes custodians of this knowledge die before passing it on [9].

To control the loss of IK related to TM it is necessary to document and conserve as much of this knowledge as possible in line with WHO policy and as reflected in the different resolutions adopted by the World Health Assembly [10-12] and WHO Regional Committee for Africa [13,14] on TM and medicinal plants. These resolutions urge Member States to among other things, produce inventories of effective practices as well as evidence on safety, efficacy and quality of traditional medicines and undertake relevant research; to take effective measures in collaboration with other partners; to ensure conservation of medicinal plants and encourage their sustainable utilization; and to respect, preserve and widely communicate, as appropriate, the IK and practices.

Previous studies in Uganda have inventoried herbal medicines and associated IK of processing and administration [3], in general ethnobotanical studies or in disease specific inventories such as those targeting malaria, tuberculosis [15], or HIV/AIDS and related conditions [16]. Owing to the wide cultural diversity and ecological diversity a high diversity of IK including that associated with herbal medicines exists in Uganda. However, little of this knowledge has been documented to date. This study complements the earlier studies by extending our knowledge of herbal medicines in four culturally and ecologically diverse regions of Uganda.

## Methods

**Figure 1:**
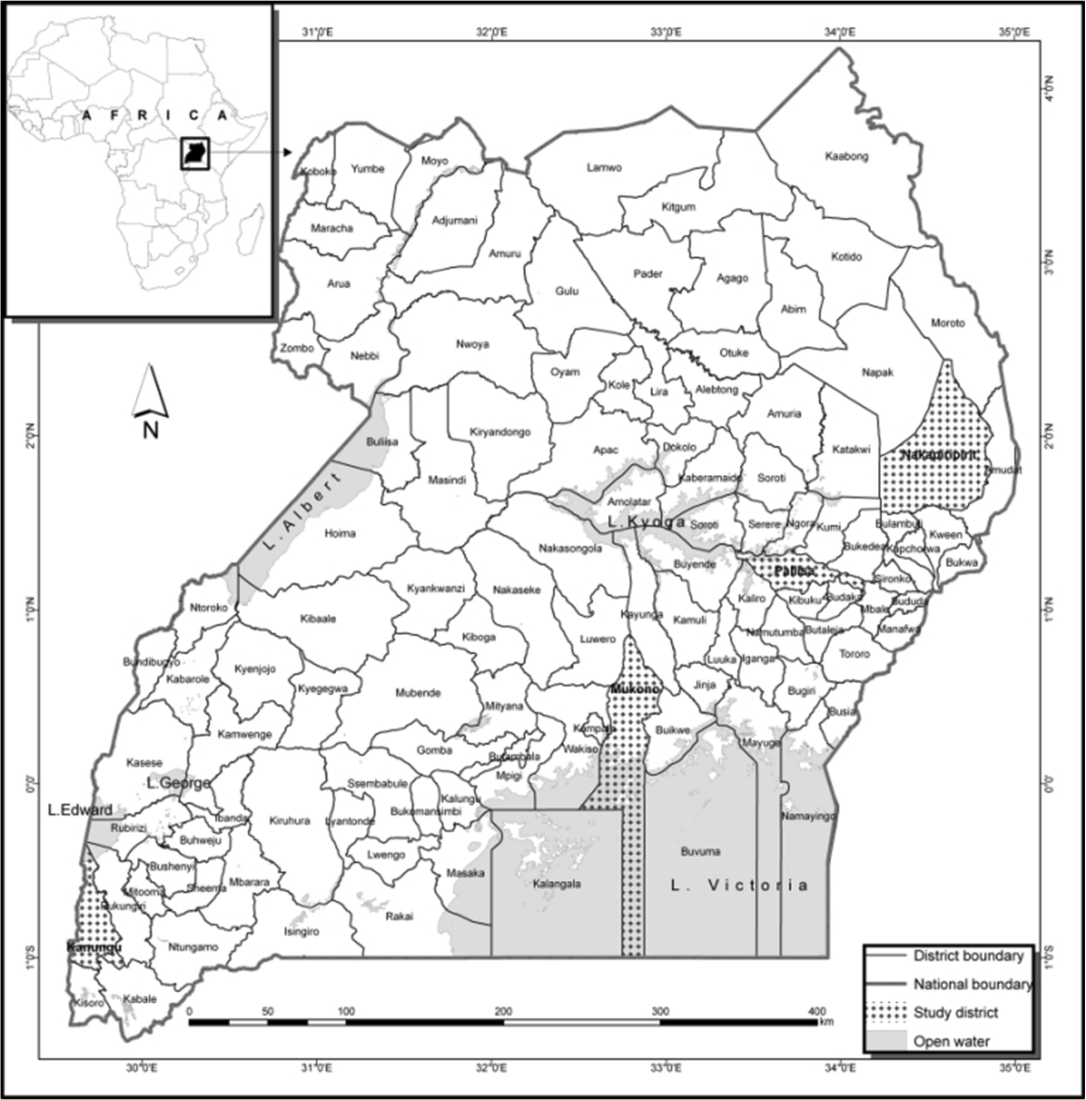
**Map of Uganda showing the four study districts, Nakapiripirit, Mukono, Kanungu and Pallisa (shaded on the map).** Inset is a map of Africa.

Field research for this project was conducted between October 2008 and February 2009 in the districts of Mukono, Nakapiripirit, Kanungu and Pallisa (Figure 1). These districts were selected because they are rural and remote with poor infrastructure and service delivery. Rural and remote communities are known to be marginalized in terms of access to health services and suffer high levels of poverty because they lack appropriate means of income generation [9]. These factors force people to use traditional medicine (TM) and keeps the indigenous knowledge associated with TM intact.

The communities in the study districts are ethnically diverse and belong to different tribes. The people of Mukono belong to the Baganda tribe. The people of Nakapiripirit are Ngakarimojong by tribe, those of Kanungu are Bakiga and the ones of Pallisa belong to the tribes of the Ateso and the Bagwere. This implies that they have different IK and exploit useful plants in different ways. All these cultural groups subsist on crop agriculture as their main source of livelihood apart from the Ngakarimajong who are nomadic cattle keepers [17].

Data were collected using an ethnobotanical survey. The survey started off with key-informant interviews that included local politicians, elderly people, and a nursing sister as participants. In the key informants interviews the focus was on understanding health seeking behavior.

The plant species used to treat ailments in the home were documented in household interviews using a questionnaire modified from that used by Almeida et al. [18]. To select respondents to participate in the interviews one village was selected serendipitously from each district. In each chosen village between 40 and 50 households were selected by simple random sampling. Interviews with children were conducted at one of the primary school in each of the villages. Ten pupils attending classes between primary 4 and 7, and equally distributed in gender were identified by the head teacher for the interviews. Altogether we interviewed 171 respondents (93 female and 78 male).

Our interviews centered on the following types of information: Common ailments that afflict households; form of health care sought by community members; specific ailments for which people seek health care from TMPs; materials used to treat ailments in traditional medicine; how the materials are used to treat ailments; and perceptions of efficacy.

In order to prioritize among the widely diverse herbal medicine plant species we conducted a Rapid Market Survey to determine the most important medicinal plants. According to Cunningham [19], highly valued species routinely appear in markets. We interviewed 20 market vendors of Owino market specialized in the sale of traditional medicines. Owino market is the largest market in Uganda. Vendors were requested to, among other things, name and rank the most valuable medicinal species. Species mentioned in all interviews were collected and identified with the help of a parataxonomist, and archived at Makerere University Herbarium (MHU). Species were identified using the Flora for Tropical Africa. Species names were verified by making reference to the IPNI (International Plant Names Index; http://www.ipni.org).

Permission to conduct this study was sought for and granted by the Uganda National Council for Science and Technology (SS 2163). In every village we requested for and acquired an endorsement to conduct the study by the local village politicians. Before every interview, the purpose, method and end use for the data collected were explained to every respondent before requesting for permission to interview the respondent.

### Data analysis

All questionnaire data was entered into Microsoft ® Office Excel and later imported into SPSS 12.0.1 for Windows, for analysis. Frequencies were summarized and percentages calculated from the data. Chi-square was computed to detect associations between the tribe and sex as independent variables and number of remedies as the dependent variable. A Pearson correlation coefficient was computed to detect the association between age and number of remedies. The number of ailments that a respondent knew how to treat was equated to richness of IK. We also calculated the informant consensus factor (ICF) to determine the disease systems where there was highest consensus on plants used in treatments. A high consensus factor (close to 1) means that the community is confident in the choice of plants, whereas a low ICF (close to 0) means that the community is still experimenting and that the treatments may not be effective [20]. Disease systems with 2 or fewer respondents were not considered when performing the ICF analysis. The ICF is calculated using the expression:

(1) $$ICF=\frac{N_{\mathrm{ur}}-T}{N_{\mathrm{ur}}-1}$$

Whereby N_ur_ represents the number of respondents mentioning a disease and, ‘T’ the number of plant species mentioned for the disease.

## Results

### Respondent characteristics

The respondents interviewed in this study had attained low levels of formal education (Table 1): the majority had attained no higher than primary level education (57%), and 23% had not attained any formal education. Most respondents were Christians (> 90%). The respondents mostly belonged to the tribes Ngakarimojong, Bakiga, Bagwere and Baganda. Their livelihoods occupations were crop farming. Others, especially those from Nakapiripirit District, were not employed in wage labor.

**Table 1:** Respondent characteristics (n = 171)

**Characteristic**	**Percent**
Education of respondent	
Primary	59
None	23
Lower secondary	11
Tertially	4
Higher secondary	2
University	1
Religion	
Catholic	61
Anglican	31
Moslem	7
Adventist	1
Yudaya	1
Tribe	
Karamojong	30
Mukiga	23
Muganda	20
Mugwere	19
Iteso	2
Musoga	1
Tanzanian	1
Munyarwanda	1
Mulundi	1
Munyole	1
Mukonjo	1
Occupation of respondent	
Farmer	42
Student	22
Pastoralist	20
Civil servant	7
Housewife	3
Business	1
Crafts Man	1
Tailor	1
Mechanic	1
Traditional Livestock Healer	1
Watchman	1

* local name or terminology.

### Indigenous knowledge of traditional medicine use

**Figure 2:**
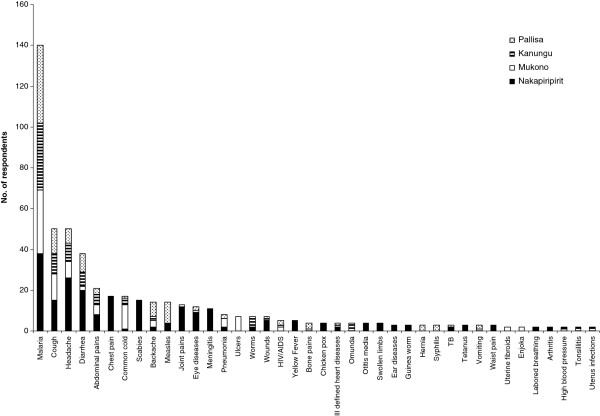
**Most frequently mentioned ailments by respondents of Nakapiripirit, Mukono, Kanungu and Pallisa.** Included are diseases mentioned by two or more respondents.

**Figure 3:**
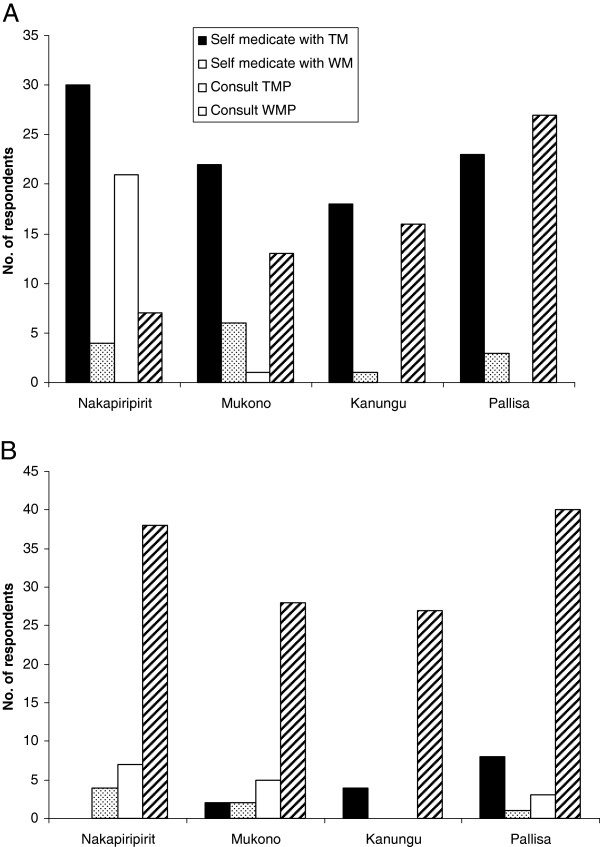
**Health seeking behaviors of respondents in Nakapiripirit, Mukono, Kanungu and Pallisa.a**. first form of health care sought. **b**. secondary form of care sought when the first provider or source fails to work.

In this study the common diseases afflicting people in the four study districts include malaria, cough, headache, diarrhea, abdominal pain, flu, back-ache and eye diseases (Figure 2). When people fall sick they either self-medicate using traditional medicines or consult western medicine practitioners (Figure 3a). Nakapiripirit is unique in this regard, because people either self medicate using traditional medicine (TM) or consult traditional medicine practitioners (TMPs). In all the studied districts, when the first form of care sought does not yield positive outcomes, people will consult western medicine practitioners (Figure 3b).

**Figure 4:**
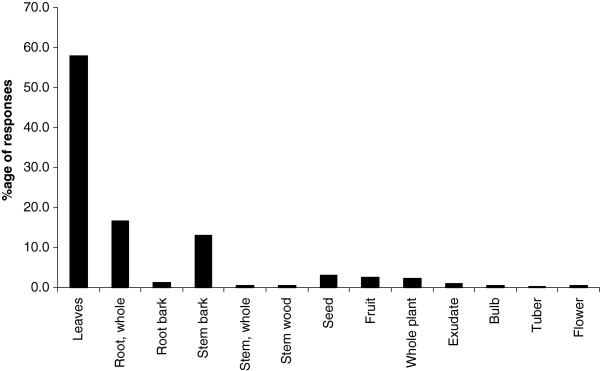
Plants parts used in the preparation of herbal medicines.

**Figure 5:**
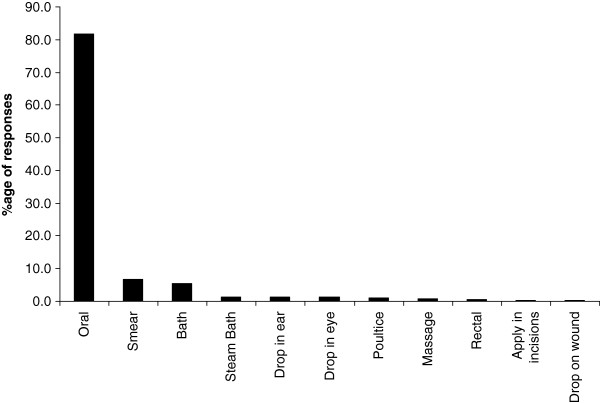
Routes of administration of traditional medicines.

**Figure 6:**
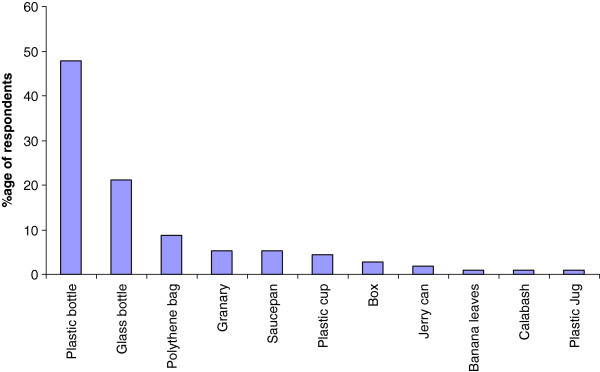
Storage practices of herbal medicines by respondents.

**Figure 7:**
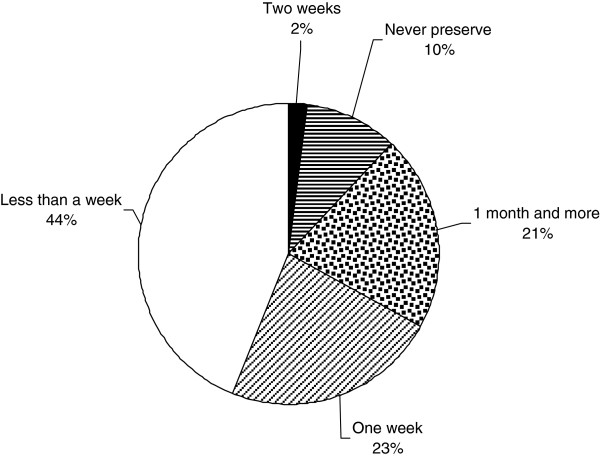
Period of storage of herbal medicines before they get spoiled.

Respondents use plants to prepare TM therapies. They infrequently add animal products, inorganic materials e.g. soil, kitchen soot and water (Table 2). The treatments are not accompanied with any rituals. On average every respondent knows how to treat at least three ailments using herbal medicines. Leaves are the most commonly used plant organ for the preparation of herbal medicines; roots and stems are also commonly used (Figure 4). The medicines are mostly prepared as water extracts or as decoctions and administered orally (Figure 5). Herbal medicines are stored in plastic bottles (Figure 6), and according to one respondent, it is only medicines prepared from difficult to find species that are stored; easy to find species are not. Medicines when stored last for short periods of time (Figure 7).

**Table 2:** Other material used in treatments

**Material**	**Ailment**
1.White chalk soil	Chicken pox
2.Animal parts (fat, offal, blood, butter and cow dung)	Chest pain
a.Milk from a black cow	Measles
b.fat	Tuberculosis
3.Ash	Febrile convulsions, Wounds, Malaria
4.Coral salt, Ebalangit	Toothache
5.Hot cloth	Headache
6.Petroleum Jelly	Pneumonia, Fractures
*7.Kihonde*	
8.Kitchen soot	Worms
9.Rabbit hair and parts of the animal called *Napupu*	Burns
10.Salt and Rock salt	Wounds
11.Anthill soil	Pyomyositis
12.Soil, Red soil,	Diarrhea, Tonsillitis, Worms, Malaria Cough, Malaria
13.Water from a grass roof	Uterine fibroids

We documented 262 plants used to treat 78 diseases and medical conditions (Table 3). 151 of these have been identified to species level. The rest of the species could not be identified because we failed to collect voucher specimens due to the prevailing insecurity in Nakapiripirit at the time this study was conducted. Nine plants, although unidentified have been included in the list because they were mentioned by three or more respondents. 44 of the species have been prioritized here on the basis of being known to treat four or more diseases/conditions (Additional file 1: Table S1). The species used to treat ailments varied among the study communities (data not shown), with the Nakapiripirit respondents mentioned the most disparate species.

**Table 3:** Species mentioned in interviews, their families and frequency of mention by respondents

**Species**	**Family**	**Freq.**
*Vernonia amygdalina* Delile	Asteraceae	82
*Aloe* sp.	Aloaceae	37
*Azadirachta indica* A. Juss.	Meliaceae	30
*Cassia nigricans* Vahl.	Caesalpiniaceae	24
*Mangifera indica* L.	Anacardiaceae	22
*Carica papaya* L.	Caricaceae	16
*Momordica foetida* Schumach.	Cucurbitaceae	15
*Chasmanthera dependens* Hochst.	Menispermaceae	11
*Acacia nilotica* (L.) Willd. ex Delile	Mimosaceae	10
*Psidium guajava* L.	Myrtaceae	9
*Senna occidentalis* (L.) Link	Caesalpiniaceae	9
*Warburgia salutaris* (G. Bertol.) Chiov.	Canellaceae	9
*Bidens pilosa* L.	Asteraceae	8
*Physalis peruviana* L.	Solanaceae	8
*Vernonia lasiopus* O. Hoffm.	Asteraceae	8
*Albizia anthelmintica* Brongn.	Mimosaceae	7
*Carissa edulis* (Forssk.) Vahl.	Apocynaceae	7
*Abrus precatorius* L.	Papilionaceae	6
*Aristolochia elegans* Mast.	Aristolochiaceae	6
*Dracaena steudneri* Engl.	Dracaenaceae	6
*Lantana camara* L.	Verbenaceae	6
*Plectranthus barbatus* Andr.	Lamiaceae	6
*Bothriocline longipes* (Oliv. & Hiern) N. E. Br.	Asteraceae	5
*Callistemon citrinus* (Curt.) Stapf.	Myrtaceae	5
*Citrus sinensis* (L.) Osbeck	Rutaceae	5
*Conyza sumatrensis* (Retz.) E. Walker	Asteraceae	5
*Crassocephalum crepidioides* (Benth.) S.Moore	Asteraceae	5
*Cymbopogon nardus* (L.) Rendle	Poaceae	5
*Indigofera arrecta* Hochst.	Papilionaceae	5
*Lantana trifolia* L.	Verbenaceae	5
*Ocimum gratissimum* L.	Lamiaceae	5
*Sida acuta* Burm. f.	Malvaceae	5
*Zanthoxylum leprieurii* Guill. & Perr.	Rutaceae	5
*Cupressus lusitanica* Mill.	Cupressaceae	4
*Digitaria abyssinica* (A. Rich.) Stapf	Poaceae	4
*Erythrina abyssinica* Lam.	Papilionaceae	4
*Eucalyptus* spp.	Myrtaceae	4
*Euphorbia tirucalli* L.	Euphorbiaceae	4
*Jatropha curcas* L.	Euphorbiaceae	4
*Leonotis nepetifolia* (L.) R. Br.	Lamiaceae	4
*Nicotiana tabacum* L.	Solanaceae	4
*Persea americana* Mill.	Lauraceae	4
*Saba comorensis* (Boj) Pich.	Apocynaceae	4
*Tagetes minuta* L.	Asteraceae	4
*Zizyphus mauritiana* Lam.	Rhamnaceae	4
*Acacia abyssinica* Hochst. ex Benth.	Mimosaceae	3
*Acacia oerfota* (Forssk.) Schweinf.	Mimosaceae	3
*Albizia coriaria* Welw. ex Oliv.	Mimosaceae	3
*Allium cepa* L.	Alliaceae	3
*Euphorbia* sp.	Euphorbiaceae	3
*Ficus natalensis* Hochst.	Moraceae	3
*Indigofera garckeana* Vatke	Papilionaceae	3
*Kigelia africana* (Lam.) Benth.	Bignoniaceae	3
*Melia azedarach* Linn.	Meliaceae	3
*Musa acuminata* Colla	Musaceae	3
*Sarcocephalus latifolius* (Smith) Bruce	Rubiaceae	3
*Triumfetta rhomboidea* Jacq.	Tiliaceae	3
*Acacia mellifera* (Vahl) Benth	Mimosaceae	2
*Acacia spirocarpa* Hochst. ex A. Rich.	Mimosaceae	2
*Ageratum conyzoides* L.	Asteraceae	2
*Artocarpus heterophyllus* Lam.	Moraceae	2
*Aspilia mossambicensis* (Oliv.) Wild	Asteraceae	2
*Canarium schweinfurthii* Engl.	Burseraceae	2
*Cardiospermum halicacabum* L.	Sapindaceae	2
*Chenopodium opulifolium* Schrad. ex Koch & Ziz	Chenopodiaceae	2
*Cissampelos mucronata* A.Rich.	Menispermaceae	2
*Citrus limon* (L.) Burm.f.	Rutaceae	2
*Coffea canephora* Pierre ex A. Froehner	Rubiaceae	2
*Commiphora africana* (A. Rich.) Engl.	Burseraceae	2
*Cynodon* spp.	Poaceae	2
*Emilia coccinea* (Sims) G. Don	Asteraceae	2
*Euphorbia heterophylla* L.	Euphorbiaceae	2
*Galinsoga parviflora* Cav.	Asteraceae	2
*Gouania longispicata* Engl.	Rhamnaceae	2
*Hibiscus fuscus* Garcke	Malvaceae	2
*Hoslundia opposita* Vahl	Lamiaceae	2
*Imperata cylindrica* (L.) P.Beauv.	Poaceae	2
*Lagenaria sphaerica* (Sond.) Naud.	Cucurbitaceae	2
*Manihot esculenta* Crantz	Euphorbiaceae	2
*Mollugo cerviana* (L.) Ser.	Molluginaceae	2
*Mollugo nudicaulis* Lam.	Molluginaceae	2
*Moringa oleifera* Lam.	Moringaceae	2
*Ocimum lamiifolium* Benth.	Lamiaceae	2
*Phyllanthus guineensis* Pax	Euphorbiaceae	2
*Sesamum indicum* L.	Pedaliaceae	2
*Sesbania sesban* (L.) Merr.	Papilionaceae	2
*Solanum giganteum* Jacq.	Solanaceae	2
*Solanum incanum* L.	Solanaceae	2
*Solanum lycopersicum* L.	Solanaceae	2
*Sphaeranthus suaveolens* DC.	Asteraceae	2
*Syzygium cumini* (L.) Skeels	Myrtaceae	2
*Terminalia brownii* Fres.	Combretaceae	2
*Tetradenia riparia* (Hochst.) Codd	Lamiaceae	2
*Ximenia americana* L.	Olacaceae	2
*Zingiber officinale* Roscoe	Zingiberaceae	2
*Acacia mearnsii* De Wild.	Mimosaceae	1
*Acacia polyacantha* Willd.	Mimosaceae	1
*Acacia* sp.	Mimosaceae	1
*Achyranthes aspera* L.	Amaranthaceae	1
*Albizia gummifera* (J.F. Gmel.) C.A.Sm.	Mimosaceae	1
*Alstonia boonei* De Wild.	Apocynaceae	1
*Asparagus racemosus* Willd.	Asparagaceae	1
*Balanites aegyptiacus* (L.) Delile	Balanitaceae	1
*Blumea alata* (D.Don) DC.	Asteraceae	1
*Bridelia micrantha* (Hochst.) Baill.	Euphorbiaceae	1
*Cajanus cajan* (L.) Millsp.	Papilionaceae	1
*Cannabis sativa* L.	Cannabinaceae	1
*Cleome gynandra* L.	Capparaceae	1
*Clerodendrum rotundifolium* Oliv.	Verbenaceae	1
*Combretum collinum* Fresen.	Combretaceae	1
*Commelina africana* L.	Commelinaceae	1
*Cucurbita pepo* L.	Cucurbitaceae	1
*Cyphostemma cyphopetalum* (Fresen.) Desc. Ex Wild & Drumm.	Vitaceae	1
*Desmodium adscendens* (Sw.) DC.	Papilionaceae	1
*Dicrocephala integrifolia* (L.f.) O. Kuntze	Asteraceae	1
*Erythrococca bongensis* Pax	Euphorbiaceae	1
*Ficus asperifolia* Miq.	Moraceae	1
*Ficus saussureana* DC.	Moraceae	1
*Gutenbergia cordifolia* Benth. ex Oliv	Asteraceae	1
*Hyptis suaveolens* (L.) Poit.	Lamiaceae	1
*Ipomoea batatas* (L.) Lam.	Convolvulaceae	1
*Justicia betonica* L.	Acanthaceae	1
*Kalanchoe densiflora* Rolfe	Crassulaceae	1
*Markhamia lutea* (Benth.) K. Schum.	Bignoniaceae	1
*Melinis repens* (Willd.) Zizka	Poaceae	1
*Melothria punctata* Cogniaux	Cucurbitaceae	1
*Microglossa pyrifolia* (Lam.) O. Ktze.	Asteraceae	1
*Myrica salicifolia* Hochst. Ex A. Rich.	Myricaceae	1
*Ocimum basilicum* L.	Lamiaceae	1
*Oxalis corniculata* L.	Oxalidaceae	1
*Oxygonum sinuatum* (Meisn.) Dammer	Polygonaceae	1
*Passiflora edulis* Sims	Passifloraceae	1
*Pennisetum purpureum* K. Schumach.	Poaceae	1
*Phytolacca dodecandra* L'Her.	Phytolaccaceae	1
*Portulaca quadrifida* L.	Portulacaceae	1
*Priva cordifolia* Druce	Verbenaceae	1
*Rhus vulgaris* Meikle	Anacardiaceae	1
*Rumex usambarensis* (Dammer) Dammer	Polygonaceae	1
*Rytigynia* spp.	Rubiaceae	1
*Saccharum officinarum* L.	Poaceae	1
*Senna didymobotrya* (Frisen.) Irwin & Barneby	Caesalpiniaceae	1
*Senna siamea* (Lam.) H.S. Irwin & Barneby	Caesalpiniaceae	1
*Sida cuneifolia* Roxb.	Malvaceae	1
*Solanum aculeastrum* Dunal	Solanaceae	1
*Solanum aethiopicum* L.	Solanaceae	1
*Steganotaenia araliacea* Hochst.	Apiaceae	1
*Tephrosia vogelii* Hook. f.	Papilionaceae	1
*Thunbergia alata* Bojer ex Sims	Acanthaceae	1
*Vigna unguiculata* (L.) Walp.	Papilionaceae	1
*Vitex fischeri* Gurke	Verbenaceae	1
*Zehneria scabra* (L.f.) Soud.	Cucurbitaceae	1
Unidentified (Tadeo)		5
Unidentified (Eyoroit)		4
Unidentified (Musuku)		4
Unidentified (Nalongo)		3
Unidentified (Ethiokan)		3
*Unidentified (Etabataba)*		*3*

Apart from a few species mentioned by three or more people, only identified species are shown.

The informant consensus factor (ICF) was highest for meningitis, scabies, *enjoka*^*a*^*,* snake bite, malaria, uterus infection, diarrhea, wounds, cough, headache, measles, fever, abdominal pain, common cold, worms and yellow fever (Table 4). The high ICF for these ailments suggests that the herbal medicines used to treat them are potentially efficacious. This analysis is well collaborated by observations reported by respondents on efficacy. That is to say five or more respondents reported that treatments for malaria, cough, headache, abdominal pain, diarrhea and meningitis were efficacious (Table 5).

**Table 4:** Informant consensus factor for diseases by respondents from Nakapiripirit, Pallisa, Kanungu, and Mukono

**Disease**	**ICF**
Meningitis	0.92
Scabies	0.92
*Enjoka*	0.88
Snake bite	0.80
Malaria	0.76
Uterus infection	0.75
Diarrhea	0.68
Wounds	0.67
Cough	0.55
Headache	0.54
Measles	0.50
Fever	0.50
Abdominal pain	0.38
Flu	0.38
Worms	0.33
Yellow fever	0.33

Only disease where 3 or more respondents mentioned herbal medicines are reported here.

**Table 5:** Perceptions on the efficacy of herbal medicines for the treatment of different diseases

**Disease**	**Improved**	**Recovered**	**some times Recovered**	**Did not improve**	**Total**
Malaria	24	26	1	1	52
Cough	7	9			16
Headache	2	5			7
Abdominal pain	1	5			6
Diarrhea	3	2			5
Meningitis	2	3			5
Flu	1	2		1	4
Abdominal worms		4			4
Joint pains	2	1			3
Dysentery	2				2
Pneumonia	1	1			2
Measles	1	1			2
Ulcer		2			2
Yellow fever	1	1			2
Anemia		1			1
Chest pain	1				1
Pyomystis		1			1
Fever				1	1
Gastritis		1			1
Headache	1				1
Lameness		1			1
Malnutrition in children		1			1
*Nkadomo*	1				1
*Obulogo*		1			1
*Omunda*	1				1
Uterus infection		1			1
Vomiting		1			1
Waist pain	1				1
Whitlow		1			1

**Figure 8:**
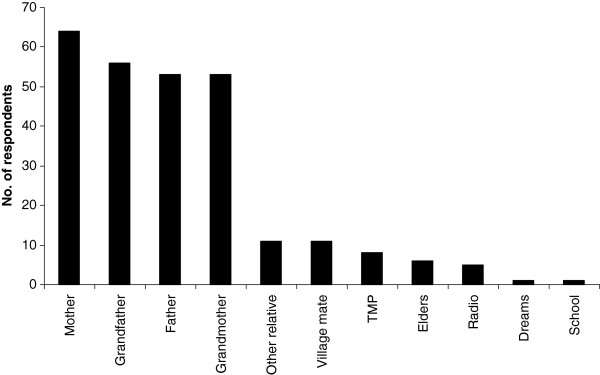
**Source of knowledge on how to use plants for healing.** Other relatives reported in the study are aunties and brother.

The knowledge to treat ailments is acquired from a variety of sources by respondents, the most important of these being parents and grandparents (Figure 8). Gender does not seem to affect level of IK associated with herbal medicine in this study (chi-sq = 3.508, p > 0.05). Age and tribe, on the other hand, affect the level of knowledge. Older people mentioned more herbal remedies than younger people (r = 0.2, p< 0.05). The people of Nakapiripirit, the Ngakarimojong, mentioned more remedies than respondents from other ethnic groups (up to six compared to three from other ethnic groups).

A survey of medicinal plants sold by market vendors revealed that most of the medicinal species mentioned in interviews in this survey were not sold in the market. Table 6 shows 35 species encountered in markets and which were mentioned by three or more respondents. Of the35 species, only 11 appear in the medicinal plants inventory reported here.

**Table 6:** The most frequently sold herbal medicine species by vendors of Owino market

**Species**	**Local name**	**Mean Rank**
Unidentified	Dokiyo	1.0
*Warburgia salutaris* (G. Bertol.) Chiov.	Abasi	1.7
Unidentified	Mbaluka	1.9
*Securidaca longipedunculata* Fres.	Mukondwe	2.0
*Mangifera indica* L.	Muyembe	2.3
*Zanthoxylum chalybeum* Engl.	Ntale ya dungu	2.3
*Psorospermum febrifugum* Spach	Kanzironziro	2.4
*Alstonia boonei* De Wild.	Mubaja ngalabi	2.5
*Piliostigma thonningii*	Mugaali	2.7
(Schumach.) Milne-Redh.		
*Garcinia buchananii* Baker	Musaali	3.2
*Vernonia amygdalina* Delile	Mululuza	3.3
*Ziziphus pubescens* Oliver	Mugenda kilo	3.4
*Entada abyssinica* A.Rich.	Mwolola	3.5
*Albizia coriaria* Welw. ex Oliv.	Mugavu	3.9
*Myrica kandtiana* Engl.	Kikimbo	4.0
*Acacia polyacantha* Willd.	Kibeere	4.2
*Syzygium cumini* (L.) Skeels	Jambula	4.3
*Toddalia asiatica* (L.) Lam.	Kawule	4.3
*Acacia hockii De Wild.*	Kasaana	4.5
*Aristolochia elegans* Mast.	Musuja welaba	4.8
*Zanthoxylum* spp.	Munyeeye	4.9
*Erythrina abyssinica* Lam.	Jirikiti	5.0
*Rhus vulgaris* Meikle	Kakwanso kwanso	5.1
*Prunus africana* (Hook.f.) Kalkman	Ntaseesa	5.1
*Spathodea campanulata* P. Beauv.	Kifabakazi	5.1
*Canarium schweinfurthii* Engl.	Muwafu	5.1
*Carissa edulis* (Forssk.) Vahl.	Muyonza	5.3
*Piptadeniastrum africanum* (Hook.f.) Brenan	Mpewere	5.4
Unidentified	Naligwalimu	5.4
*Cryptolepis sanguinolenta* (lindl.) Schltr.	Kafulu	5.8
*Kigelia africana* (Lam.) Benth.	Mussa	6.3
*Combretum molle* G.Don	Ndagi	6.5
Unidentified	Muwo	6.7
*Dracaena steudneri* Engl.	Kajjolyenjovu	7.7
*Albizia* spp.	Nongo	8.3

Mean ranks are also shown. A rank of 1 shows a species known to be the most important and 8 the least important among the sold species. Only species mentioned by 3 or more vendors are shown. Species mentioned by respondents in household interviews are highlighted.

## Discussion

Respondents interviewed in this survey have knowledge to treat 78 ailments and conditions. Herbal medicine knowledge is extensive as every respondent can, on average, mention three remedies. However, this knowledge varied from district to district. It was not possible to do a complete review of the ethnomedicinal uses of the species reported here. However, and at least for the species reported here as being the most commonly used to treat malaria, similar therapeutic claims have been reported from other parts of the world (Table 7). Furthermore, for some of the species like *Azadirachta indica* antiplasmodial activity has also been demonstrated in vivo and in vitro [21]. The similar use of a plant species for the treatment of the same ailment (like malaria in this case) in different regions of the world is one form of evidence that the species in question may be efficacious and also safe to use [22,23]. For the species shown in Table 7, therefore, there appears to be evidence that the species are effective in treating malaria.

**Table 7:** Comparative ethnomedicinal use for the treatment of malaria in other studies and reports of antiplasmodial activity

**Species**	**Reference**
*Vernonia amygdalina*	[26], references in [26], [27]. Antiplasmodial activity is reported by Tona et al. [28]
*Aloe spp.*	[29], [27]
*Azadirachta indica*	[26], references in [26]. Antiplasmodial activity is reported in references by Nguta et al. [29] and Sofowora [21], and in a review by Soh and Benoit-Vical [30], [27]
*Mangifera indica*	[26], references in [26], [29]

In this study respondents were seen to self medicate using plants and allopathic medicines. Herbal medicines are frequently used in self medication to alleviate symptoms or shorten recovery time in self limiting ailments^b^[22], such as malaria and diarrhea. On the other hand, allopathic medicine is preferred for the treatment of serious diseases and conditions like tuberculosis.

The practice of self-medication is popular in many parts of the world including Africa where the health infrastructure is poor, or where people have a negative attitude about the quality of care in medical facilities, or people can ill afford the consultation fees charged in medical facilities [13,24,25]. Respondents in this study stated that TMPs are not usually consulted (except in Nakapiripirit the most marginalized of the study districts). According to Tabuti et al. [3], TMPs are commonly consulted for chronic and difficult to understand ailment. The implications of poor patronage of TMP may be a faster loss of IK associated with TM.

Knowledge of how to treat ailments by respondents is acquired from parents and grandparents. Indeed older people mention significantly more remedies than the young. This is in agreement with studies conducted elsewhere which show that older people have more IK than younger ones and that they are the ones who transmit this knowledge [9].

## Conclusions and recommendations

This survey has indicated that abundant indigenous knowledge on traditional medicine (TM) still exists and that TM is still important in Uganda, because respondents mentioned many species and remedies used in traditional medicine and stated that they use it as a first line of health care when they fall sick. The patronage of TMPs in this study appears to be low. This is somewhat confusing given the reportedly important role that TMPs play in TM [4]. More rigorous health seeking behavior studies should be conducted to clarify this important aspect of TM.

There is need to validate the efficacy and safety of the remedies reported in this study to determine whether they are effective to treat the diseases that they are claimed to treat and are safe to use. Priority species should be those that are used to treat diseases that respondents have observed to be effective and for which people have the highest agreement measured as informant consensus factor (ICF) such as meningitis (Additional file 1: Table S1). The first step in this direction should be to conduct a literature review to see what information exists for the target species before bioassays are conducted in the laboratory.

Secondly, this study was conducted in only four districts of Uganda, and showed that IK varied between districts. This means that more detailed surveys covering the whole country remain to be undertaken to complete the documentation of this knowledge in Uganda. Thirdly, a wider market survey of medicinal plants needs to be conducted to capture the whole diversity of herbal medicine species sold in markets.

## Endnotes

^a^Enjoka is an all inclusive term that may refer to abdominal worms. It may also mean abdominal cramps/pain which are believed to be caused by worms among other things; gonorrhea; or painful menses in females

^b^Self limiting ailments are disease conditions whose symptoms may disappear even without treatment

## Competing interests

The authors declare that they have no competing interests.

## Authors' contributions

JRST and DK conceived the study, and participated in its design and coordination. All authors helped to draft the manuscript, read and approve the final manuscript.

## Supplementary Material

Additional file 1 **Table S1.** The most commonly used herbal medicine plants and the diseases that they treat. Click here for file
